# Short- and long-term evaluation of disease-specific symptoms and quality of life following uterine artery embolization of fibroids

**DOI:** 10.1186/s13244-022-01244-1

**Published:** 2022-06-21

**Authors:** Iason Psilopatis, Florian Nima Fleckenstein, Federico Collettini, Elif Can, Anne Frisch, Bernhard Gebauer, Uli Fehrenbach, Giovanni Federico Torsello, Dirk Schnapauff, Matthias David, Gero Wieners

**Affiliations:** 1 Department of Diagnostic and Interventional Radiology, Charité – Universitätsmedizin Berlin, Corporate Member of Freie Universität Berlin and Humboldt-Universität zu Berlin, Augustenburger Platz 1, 13353 Berlin, Germany; 2 Department of Gynecology, Charité – Universitätsmedizin Berlin, Corporate Member of Freie Universität Berlin and Humboldt-Universität zu Berlin, Berlin, Germany; 3 Berlin Institute of Health at Charité – Universitätsmedizin Berlin, BIH Biomedical Innovation Academy, BIH Charité Clinician Scientist Program, Berlin, Germany; 4 Radiologische Praxis am Kapweg, Berlin, Germany

**Keywords:** Quality of life, Uterine leiomyoma, UFS-QoL questionnaire, Response to treatment, Interventional radiology

## Abstract

**Background:**

The purpose of this study is to evaluate uterine artery embolization (UAE) for the management of symptomatic uterine leiomyomas regarding changes in quality of life after treatment in a large patient collective. This study retrospectively analyzed prospectively acquired standardized questionnaires of patients treated with UAE. Clinical success was evaluated before and after embolization. Patients were stratified into short- (≤ 7 months) and long-term (> 7 months) follow-up groups depending on the time of completion of the post-interventional questionnaire. Uterine leiomyomas were furthermore divided into small (< 10 cm) and large (≥ 10 cm) tumors based on the diameter of the dominant fibroid.

**Results:**

A total of 245 patients were included into the final data analysis. The Kaplan–Meier analysis showed a cumulative clinical success rate of 75.8% after 70 months until the end of follow-up (9.9 years). All questionnaire subscales showed a highly significant clinical improvement from baseline to short- and long-term follow-up (*p* < 0.001). Patients with small fibroids showed a significantly better response to UAE in multiple subcategories of the questionnaire than patients with fibroids ≥ 10 cm who had a twofold higher probability of re-intervention in the Cox-regression model.

**Conclusions:**

UAE is an effective treatment method for symptomatic fibroids that leads to quick relief of fibroid-related symptoms with marked improvement of quality of life and is associated with a low risk for re-interventions. Patients with small fibroids tend to show a better response to UAE compared to patients with large fibroids.

*Trial registration* Charité institutional review board, EA4/167/20. Registered 27 November 2020—Retrospectively registered. https://ethikkommission.charite.de/

**Supplementary Information:**

The online version contains supplementary material available at 10.1186/s13244-022-01244-1.

## Key points

- Uterine artery embolization (UAE) is an effective treatment method with a low risk for re-interventions.
- UAE leads to a quick relief of fibroid-related symptoms.
- UAE contributes to a highly significant improvement of quality of life.
- Patients with small fibroids show a better treatment response than large fibroids.

## Background

Uterine leiomyomas, also known as fibroids, are the most common benign tumors of the female genital tract in women of reproductive age [1]. Their incidence increases with age and they usually regress after menopause [2]. Although most women are asymptomatic, fibroids can cause numerous severe conditions such as dysmenorrhea with or without abnormal uterine bleeding, disruption of surrounding pelvic structures and fertility problems [3]. Besides physical impairment, the impact of symptomatic fibroids on mental health and quality of life of affected women must not be underestimated [4]. The standard treatment approach for symptomatic women consists of surgical myomectomy and hysterectomy associated with good symptom control and high patient satisfaction [5]. However, the management of symptomatic fibroids is challenging and interdisciplinary concepts are needed to offer individual therapy options. Uterine artery embolization (UAE) has proven to be an effective minimally invasive treatment alternative for patients with symptomatic fibroids [6]. In comparison with surgery, UAE has several advantages including preservation of the uterus, the use of conscious sedation rather than general anesthesia, low risk of blood loss and a shorter recovery time [7, 8].

Several short- and long-term studies have highlighted the effectiveness and safety of UAE in the reduction of clinical symptoms and improvement of Health-Related Quality of Life (HRQoL) [9–14]. Nevertheless, only few studies incorporated validated outcome measures for assessing short- and long-term changes in symptoms and HRQoL.

The use of the Uterine Fibroid Symptom and Quality of Life (UFS-QoL) questionnaires, presented by Spies et al. in 2002 [15], allows the uniform and objective assessment of disease-specific symptom severity and impairment of patient’s HRQoL before and after treatment. Standardized questionnaires facilitate the comparison with the results of similar studies incorporating validated outcome measures.

This study assessed effects of UAE on quality of life in a large patient cohort by analyzing results from standardized UFS-QoL questionnaires. The comprehensive analysis focuses on short- and long-term continuous clinical effectiveness of UAE, re-intervention rates, as well as response to treatment depending on lesion size.

## Methods

### Study setting and patient population

Between January 2010 and December 2019, 501 patients with symptomatic fibroids underwent UAE at our institution. After clinical examination and confirmation of the diagnosis by transvaginal ultrasound, imaging was completed using contrast-enhanced magnetic resonance imaging (MRI) of the pelvis. Prior to treatment, patients were seen by a gynecologist and interventional radiologists, and treatment decisions were made in interdisciplinary consent. Indication for UAE treatment was (1) over one year duration of fibroid-related symptoms and (2) failure of conservative treatment. Exclusion criteria were suspicion of malignancy, evident uterine adenomyosis, urogenital infections and general contraindications for invasive angiography. All patients included in this study gave written informed consent to undergo UAE and volunteered to participate in the follow-up examinations. Patient data analysis was performed with respect to the EU General Data Protection Regulation (GDPR) and the Good Scientific Practice of Charité-University Medicine Berlin. The study including prospective data collection was approved by the institutional review board (EA4/167/20). Permission to use the UFS-QoL questionnaire in an official German translation was granted by its authors and the Society of Interventional Radiology.

### Baseline MRI

All patients underwent MRI on a 1.5-Tesla superconducting scanner (Magnetom Vision^®^, Magnetom Symphony^®^, Siemens Medical Systems, Erlangen, Germany) using a torso phased-array coil before UAE. The standardized MRI protocol consisted of T2-weighted sequences of axial, coronal and sagittal planes, and axial T1-weighted fat-saturated sequences. Contrast-enhanced MR-angiography was performed after weight adapted intravenous infusion of gadolinium-based contrast agent (Gadovist^®^; Mississauga, Ontario, Canada) at a rate of 2 mL/s. Postcontrast fat-saturated T1-weighted sequences of axial, coronal and sagittal planes were also obtained. Uterine volume, longest diameter and localization of the dominant fibroid as well as the number of lesions were determined. Based on the longest axis of the dominant fibroid, patients were divided into two groups, as proposed in the literature [16]. The first group included patients with a dominant tumor with a longest axial diameter of at least 10 cm, while the second group included patients with smaller tumors.

### Procedure

**Fig. 1:**
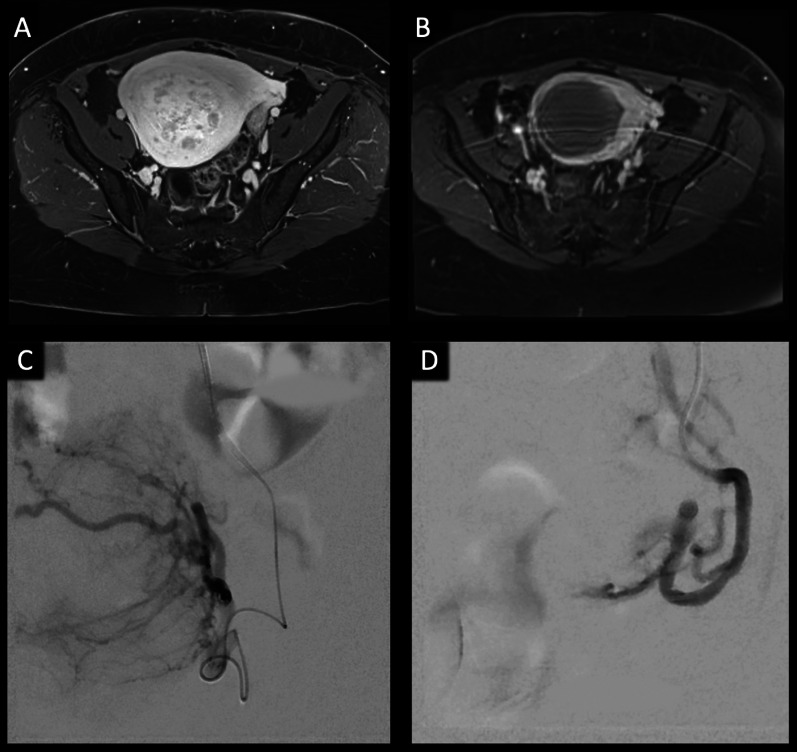
UAE. T1-weighted contrast-enhanced MRI of the pelvis before (**A**) and after (**B**) UAE in a patient showing a good response to treatment. The hypointense areas in the center of the tumor mass indicate tumor necrosis. Digital subtraction angiography of the uterine artery before (**C**) and after (**D**) embolization showing stasis in the uterine artery

All procedures were performed by board-certified radiologists with > 10 years of experience. After local anesthesia with lidocaine 1%, vascular access was established preferably in the right common femoral artery and the tip of a 4- to 5-F guiding catheter (RIM catheter; Merit Medical, Paris, France or Roberts uterine catheter; Cook, Bloomington, Ind, USA) was placed into the internal iliac artery via a 0.035-inch guide wire. After initial angiography, the tip of a 3-F microcatheter (Renegade Hi-Flo Microcatheter; BostonScientific, Natick, MA, USA) was directed into the horizontal segment of the uterine artery well beyond angiographically visualized cervicovaginal branches. As previously reported in the literature, embolization was accomplished using Trisacryl Gelatin Microsphere (TGM) particles (EmboSphere; Merit Medical, Paris, France) as well as non-spherical polyvinyl alcohol (PVA) particles (Contour; BostonScientific, Natick, Massachusetts, USA) [17]. To achieve complete fibroid infarction, bilateral embolization was performed in all patients, except for rare cases where fibroids were supplied by only one side, thus allowing for a unilateral approach. According to our institution’s SOP, the angiographic endpoint of embolization was the occlusion of the peri-fibroid plexus and complete elimination of fibroid perfusion with preservation of weak antegrade flow in both uterine arteries (Fig. 1). UAE was performed as an inpatient procedure, and all patients were admitted for further observation and sufficient analgesia with intravenous narcotics if needed on the day of the intervention. Non-steroidal anti-inflammatory drugs and, in cases of severe pain, opioid derivatives were prescribed according our institution’s pain management guidelines [18].

### Clinical assessment

Pre-interventional UFS-QoL questionnaires were completed prospectively by all women at least 24 h prior to UAE to survey baseline data. All women were then asked to fill out the questionnaires after treatment during the follow-up period. Patients were stratified into short- (< 7 months) and long-term (> 7 months) follow-up groups depending on the time of completion of the post-interventional UFS-QoL questionnaire. The questionnaire includes eight questions regarding the type and severity of symptoms, that are summarized in a ‘symptom severity’ scale, as well as 29 questions on how the disease impairs several aspects of patient’s HRQoL. These were subdivided into six subscales pertaining to ‘concern,’ ‘activities,’ ‘energy/mood,’ ‘control,’ ‘self-consciousness’ and ‘sexual function’ and together represent the ‘HRQoL total score.’ A total of eight scores summarizes the results of the questionnaire that refers to the three preceding months. Patients were asked to score their symptoms (questions 1–8) on a 5-point Likert scale ranging from ‘not at all’ (1) to ‘a very great deal’ (5), and the impairment of their HRQoL (questions 9–37) from ‘none of the time’ (1) to ‘all of the time’ (5). Full UFS-QoL questionnaire as well as the German translation can be found in the supplement data (Additional file 1: S1 and S2). The formula suggested by the authors of the questionnaire was used to calculate the symptom severity score, with higher score values indicating a higher symptom severity. An inverted formula was used to calculate the HRQoL score with higher scores indicating a better HRQoL and lower scores a deterioration in HRQoL [15]. According to the UFS-QoL manual, a subscale score could not be considered for further analysis if more than 50% of the respective questions remained unanswered. Otherwise, the missing value was imputed by the mean of the other subscale values. Any surgical or endovascular intervention to control complications or persistent fibroid-related symptoms following embolization was recorded during follow-up. Treatment failure was defined as the (unplanned) need for re-intervention (surgery or re-embolization).

### Statistical analysis

Statistical analysis was performed using the SPSS software package (SPSS 26.0; SPSS Corporation, Chicago, IL, USA). Kaplan–Meier analysis was used to determine the cumulative rate of treatment failure and the mean freedom from treatment failure during the follow-up period for the entire cohort. Cox proportional hazard regression analysis was employed to look for interrelations between the tumor size, adjusted for patients’ age and the clinical outcome in terms of treatment failure. According to nonparametric distribution, the results of each UFS-QoL subscale before and after UAE are given as median with 25th and 75th percentile and are illustrated graphically. Changes within each score were tested for significance using Wilcoxon test for paired samples. Small versus large fibroid patient groups were compared using the Mann–Whitney U test for unpaired samples to detect differences in clinical improvement assessed with the UFS-QoL questionnaire between both groups before and after UAE. *p* values < 0.05 were considered statistically significant.

## Results

**Fig. 2:**
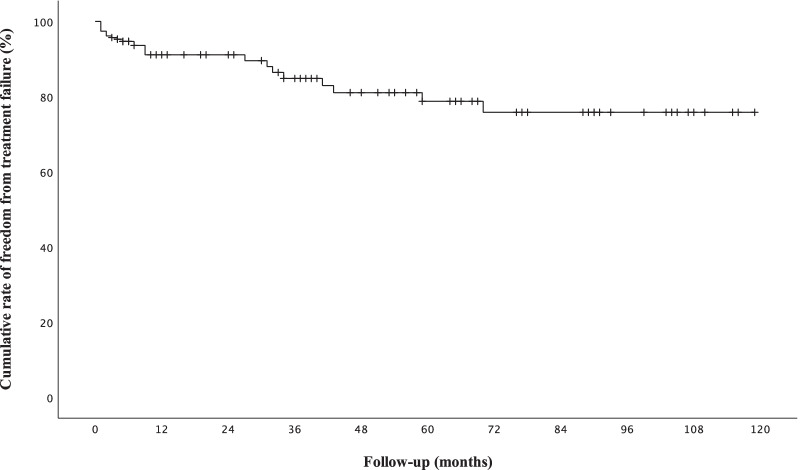
Cumulative rate of freedom from treatment failure according to Kaplan–Meier analysis

**Fig. 3:**
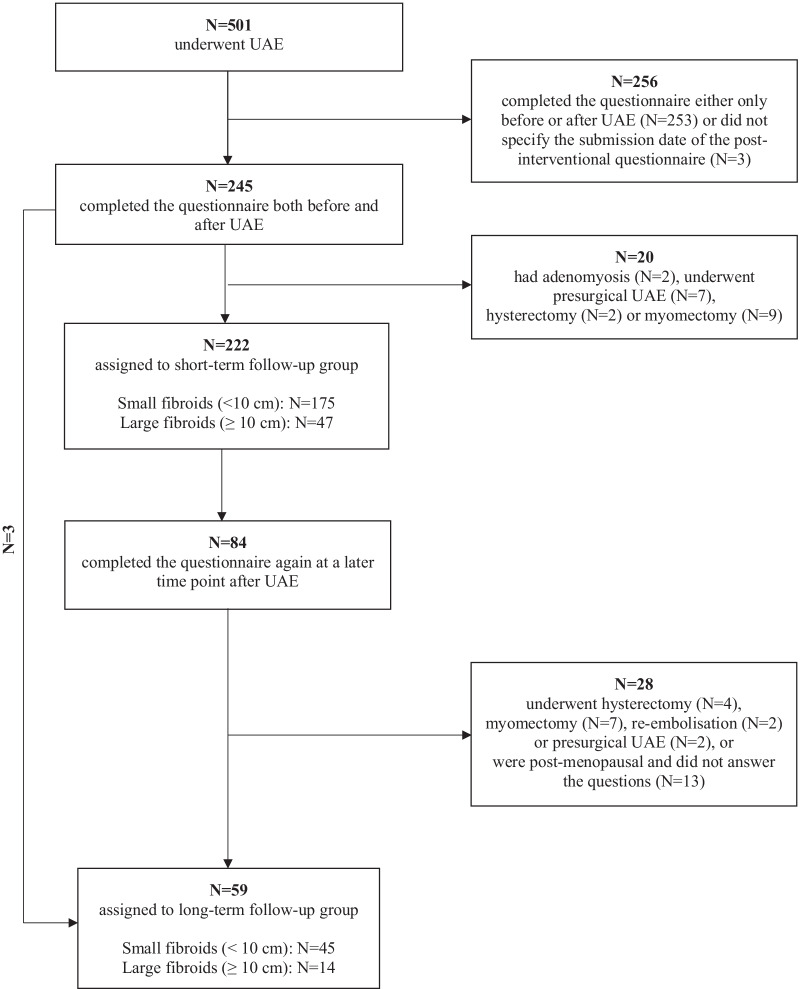
Flow diagram of patients included and excluded over the course of the study

Out of 501 patients, 245 (49%) completed the UFS-QoL questionnaire both before and at least once after UAE. Of these, two patients showed signs of adenomyosis on MRI and nine patients underwent a presurgical UAE in a time interval of hours or a few days before planned myomectomy. Twenty-two women underwent either myomectomy or hysterectomy after treatment failure. Two patients repeated the UAE treatment, while 13 women had entered menopause at the time of follow-up and were also excluded. The cumulative rate of freedom from treatment failure according to Kaplan–Meier analysis was 75.8% [95% Confidence Interval (CI), 65.0–86.6] 70 months after UAE (Fig. 2). Median patient age was 46 (25th–75th percentile, 43–49 years; range, 26–67 years). Cox regression analysis, adjusted for patients’ age, revealed that the risk of treatment failure was twice as high in women with large fibroids compared to women with small fibroids (*p* = 0.049). The remaining patients were included in the analysis of the UFS-QoL questionnaire and formed the short- (*n* = 222) and long (*n* = 59)-term follow-up groups. The short-term follow-up group included 175 patients with small and 47 patients with large fibroids, while the long-term follow-up group consisted of 45 women with small and 14 women with large fibroids (Fig. 3).

### Analysis of the UFS-QoL questionnaire

The clinical short-term outcome was assessed at a median of five months and the long-term outcome at a median of 59 months after UAE. From baseline to short-term follow-up, the ‘symptom severity’ score significantly decreased from a median of 50.0 (Quartile Range (QR), 38.3–62.5) to 21.9 (QR, 12.5–34.4; *p* < 0.001), whereas the ‘HRQoL total score’ showed a significant increase from a median of 52.6 (QR, 39.6–68.5) to 84.5 (QR, 67.9–93.1; *p* < 0.001). The most pronounced change was seen for the ‘activity’ subscale with a median increase of 35.7 points. Table 1 presents the UFS-QoL questionnaire scores after the short-term follow-up for the paired samples.

**Table 1:** Changes in symptom severity and HRQoL scores after short-term follow-up (*n* = 222)

UFS-QoL subscale	*n*^1^	Before UAE Median (25. 75.P)	Short-term follow-up Median (25. 75.P)	*p* value (Wilcoxon test)
Symptom severity	205	50.0 (38.3 62.5)	21.9 (12.5 34.4)	< 0.001
Concern	203	50.0 (30.0 80.0)	80.0 (65.0 100.0)	< 0.001
Activities	205	50.0 (32.1 67.9)	85.7 (66.1 96.4)	< 0.001
Energy/mood	205	50.0 (35.7 69.6)	78.6 (64.3 96.4)	< 0.001
Control	205	50.0 (35.0 75.0)	85.0 (65.0 95.0)	< 0.001
Self-consciousness	205	66.7 (41.7 83.3)	91.7 (66.7 100.0)	< 0.001
Sexual function	193	50.0 (25.0 75.0)	75.0 (62.5 100.0)	< 0.001
HRQoL total score	189	52.6 (39.6 68.5)	84.5 (67.9 93.1)	< 0.001

(1) *n* = Number of patients with sufficient answered questions for the calculation of transformed scores using the ‘UFS-QoL Scoring Manual’

The long-term follow-up data showed a significant improvement in comparison with the baseline values, likewise. The ‘symptom severity’ score significantly decreased from a median of 50.0 (QR, 34.4–62.5) to 9.4 (QR, 0.0–24.2; *p* < 0.001), and the ‘HRQoL total score’ showed a significant improvement from a median of 49.1 (QR, 38.8–60.3) to 95.3 (QR, 81.0–100.0; *p* < 0.001). The most prominent change during the long-term follow-up occurred in the ‘sexual function’ subscale with a median increase of 40.6 points. Table 2 presents the UFS-QoL questionnaire scores after the long-term follow-up for the paired samples.

**Table 2:** Changes in symptom severity and HRQoL scores after long-term follow-up (*n* = 59)

UFS-QoL subscale	*n*^1^	Before UAE Median (25. 75.P)	Long-term follow-up Median (25. 75.P)	*p* value (Wilcoxon test)
Symptom severity	56	50.0 (34.4 62.5)	9.4 (0.0 24.2)	< 0.001
Concern	55	55.0 (35.0 80.0)	100.0 (80.0 100.0)	< 0.001
Activities	57	46.4 (28.6 57.1)	100.0 (82.1 100.0)	< 0.001
Energy/mood	57	46.4 (30.4 71.4)	100.0 (78.6 100.0)	< 0.001
Control	58	50.0 (30.0 71.2)	100.0 (80.0 100.0)	< 0.001
Self-consciousness	58	58.3 (33.3 85.4)	100.0 (75.0 100.0)	< 0.001
Sexual function	55	37.5 (25.0 75.0)	100.0 (75.0 100.0)	< 0.001
HRQoL total score	51	49.1 (38.8 60.3)	95.3 (81.0 100.0)	< 0.001

(1) *n* = Number of patients with sufficient answered questions for the calculation of transformed scores using the ‘UFS-QoL Scoring Manual’

Of the 59 patients forming the long-term follow-up group, 56 patients had been also included in the short-term follow-up group, given that they had filled out the UFS-QoL questionnaire twice. The results at short- and long-term follow-ups were compared, revealing a significant improvement of all but two subscale scores (‘self-consciousness’ and ‘sexual function’) from short to long term. Changes between the short- and long-term assessment are displayed in Table 3.

**Table 3:** Changes in symptom severity and HRQoL scores after UAE between short- and long-term follow-up (*n* = 56)

UFS-QoL subscale	*n*^1^	Short-term follow-up Median (25. 75.P)	Long-term follow-up Median (25. 75.P)	*p* value (Wilcoxon test)
Symptom severity	55	18.7 (9.4 31.2)	9.4 (0.0 21.9)	0.002
Concern	54	85.0 (65.0 100.0)	100.0 (85.0 100.0)	< 0.001
Activities	55	87.5 (75.0 100.0)	100.0 (89.3 100.0)	0.002
Energy/mood	55	89.3 (78.6 100.0)	100.0 (82.1 100.0)	0.001
Control	55	90.0 (75.0 100.0)	100.0 (87.5 100.0)	0.015
Self-consciousness	55	91.7 (75.0 100.0)	100.0 (75.0 100.0)	0.391
Sexual function	50	93.7 (75.0 100.0)	100.0 (84.4 100.0)	0.265
HRQoL total score	49	87.1 (76.7 95.5)	98.3 (86.2 100.0)	0.001

(1) *n* = Number of patients with sufficient answered questions for the calculation of transformed scores using the ‘UFS-QoL Scoring Manual’

### Clinical efficacy in patients with small versus patients with large fibroids

Patients with small fibroids showed a statistically significant improvement in all UFS-QoL scores from baseline to short- and long-term follow-up (*p* < 0.001, respectively). A further significant improvement could be demonstrated from short- to long-term follow-up in all subscale scores, except for the ‘self-consciousness’ and the ‘sexual function’ subscales. Of note, all subscale scores significantly improved from baseline to short- and long-term follow-up in patients with large fibroids, as well. The only exception was the ‘concern’ subscale showing a trend but failing to reach statistical significance from a median of 75.0 (QR, 50.0–95.0) at baseline to 85.0 (QR, 65.0–100.0; *p* = 0.063) at short-term follow-up. From short- to long-term follow-up, a significant improvement was detected in the ‘concern,’ ‘activities’ and ‘HRQoL total score’ subscale scores.

As presented in Table 4, patients with small fibroids showed a significantly greater improvement in several UFS-QoL subscales after treatment, compared to women with large fibroids.

**Table 4:** Changes in symptom severity and HRQoL scores after UAE in patients with small versus patients with large fibroids

UFS-QoL subscale	*n*^1^	Score improvement after UAE- Small fibroids Median (25. 75.P)	Score improvement after UAE- Large fibroids Median (25.75.P)	*p* value (Mann–Whitney *U* test)
*Changes in symptom severity and HRQoL scores after short-term follow-up in patients with small versus patients with large fibroids (n* = *222)*				
Symptom severity	205	− 26.6 (− 39.4 − 12.5)	− 15.6 (− 25.0 − 4.2)	0.008
Concern	203	22.5 (3.7 45.0)	5.0 (− 10.0 20.0)	0.001
Activities	205	28.6 (9.8 46.4)	14.3 (1.8 33.9)	0.011
Energy/mood	205	21.4 (10.7 42.9)	21.4 (4.5 34.8)	0.107
Control	205	25.0 (10.0 40.0)	20.0 (8.1 40.0)	0.453
Self-consciousness	205	16.7 (0.0 33.3)	25.0 (0.0 37.5)	0.385
Sexual function	193	25.0 (0.0 50.0)	12.5 (0.0 43.7)	0.460
HRQoL total score	189	25.0 (9.7 39.6)	16.5 (5.4 29.1)	0.044
*Changes in symptom severity and HRQoL scores after long-term follow-up in patients with small versus patients with large fibroids (n* = *59)*				
Symptom severity	56	− 34.4 (− 53.1 − 23.7)	− 32.2 (− 37.5 − 11.7)	0.112
Concern	55	35.0 (10.0 60.0)	25.0 (0.0 37.5)	0.160
Activities	57	46.4 (35.7 60.7)	25.0 (8.9 44.9)	0.003
Energy/mood	57	39.3 (25.0 57.1)	25.0 (12.5 33.9)	0.027
Control	58	40.0 (20.0 57.8)	30.0 (12.8 40.0)	0.082
Self-consciousness	58	33.3 (8.3 50.0)	8.3 (0.0 27.1)	0.046
Sexual function	55	37.5 (0.0 62.5)	25.0 (12.5 37.5)	0.497
HRQoL total score	51	43.3 (25.2 52.8)	25.0 (13.1 32.5)	0.005

UFS-QoL subscale	*n*^1^	Score improvement from short- to long-term Small fibroids Median (25. 75.P)	Score improvement from short- to long-term Large fibroids Median (25. 75.P)	*p* value (Mann–Whitney *U* test)
*Changes in symptom severity and HRQoL scores after UAE between short- and long-term follow-up in patients with small versus patients with large fibroids (n* = *56)*				
Symptom severity	55	− 6.2 (− 21.9 6.2)	− 10.0 (− 27.6 2.3)	0.588
Concern	54	5.0 (0.0 20.0)	15.0 (0.0 32.5)	0.099
Activities	55	5.4 (0.0 12.5)	5.4 (0.0 16.5)	0.689
Energy/mood	55	3.6 (0.0 14.3)	0.0 (0.0 8.9)	0.245
Control	55	5.0 (0.0 15.0)	0.0 (0.0 8.7)	0.333
Self-consciousness	55	0.0 (0.0 8.3)	0.0 (0.0 3.1)	0.930
Sexual function	50	0.0 (0.0 18.7)	0.0 (0.0 6.2)	0.957
HRQoL total score	49	6.0 (0.0 11.2)	3.0 (0.0 10.8)	0.660

(1) n = Number of patients with sufficient answered questions for the calculation of transformed scores using the ‘UFS-QoL Scoring Manual’

## Discussion

This comprehensive study has three main findings: (1) UAE is an effective treatment of symptomatic fibroids. Treated women show a significant improvement of HRQoL, as measured by the standardized UFS-QoL questionnaire, and re-intervention rates are low, even years after treatment. (2) HRQoL of treated women improves significantly early after treatment and keeps improving after time with the best results in long-term follow-up. (3) Patients with small symptomatic fibroids respond significantly better to UAE when compared to women with larger fibroids. UFS-QoL scores were significantly higher and the risk of re-intervention half as low as in the large-fibroids group.

UAE plays an important role in the treatment of symptomatic fibroids in today’s clinical routine with numerous studies reporting the effectiveness and safety of this minimal-invasive approach [9–14, 19–25]. Many of these studies have focused on the rate of hysterectomies, myomectomies, or re-embolization following UAE, as the need for re-intervention is considered an objective treatment failure. Multiple studies have shown that UAE is associated with a low rate of re-interventions of approximately 15–25% in the long-term after a median of five to nine years post-intervention [10, 13, 21–23]. These results are in line with the low re-intervention rate in this study cohort (11%;* n* = 245).

Several studies report a significant improvement in ‘symptom severity,’ subcategories of HRQoL and ‘HRQoL total score’ shortly after UAE. One study reported a median ‘symptom severity’ score of 14.1 and a median ‘HRQoL total score’ of 91.8 points for the short-term follow-up group (2.5–7.5 months) [20]. Other studies including the Fibroid Registry for Outcomes Data (FIBROID) study, which evaluated UFS-QoL questionnaires of more than 1700 patients, report similar findings [12, 26, 27]. The scores obtained in the hereby presented study are consistent with the published data and achieve similar scores with a median ‘symptom severity’ score of 21.9 and a median ‘HRQoL total score’ of 84.5 points.

For long-term follow-up, the lowest possible scores are obtained for the ‘symptom severity’ and the highest possible scores for the subcategories of HRQoL and the ‘HRQoL total score.’ One study reported a median ‘symptom severity’ score of 3.13 and a median ‘HRQoL total score’ of 100.0 points 5.7 years after UAE [10], with similar studies showing similar long-term UFS-QoL scores, respectively [9, 20]. The post-intervention scores in the long-term follow-up group reported in this study, with a median ‘symptom severity’ score of 9.4 and a median ‘HRQoL total score’ of 95.3 points, are consistent with the previously reported data in the literature.

Longitudinal post-interventional improvements in UFS-QoL categories from short- to long-term follow-up are reported in several studies that surveyed patients at different time points after UAE. One study demonstrated a significant improvement in the UFS-QoL categories of ‘symptom severity,’ ‘concern,’ ‘control,’ ‘sexual function’ and ‘HRQoL total score’ over a timeframe of eight months to 6.3 years [26]. The ‘symptom severity’ score and ‘HRQoL total score’ calculated in the FIBROID study improved steadily over time [9]. The patient population in this study showed also a highly significant improvement in almost all UFS-QoL categories from short- to long-term after UAE, confirming the hypothesis that the overall confidence of treated women increases over time [28].

A large number of studies investigated whether patients with large fibroids have a worse response to UAE than patients with small fibroids. On the one hand, some authors believe that small fibroids are associated with a better response to therapy after UAE [9, 21]. Multivariable analysis on the impact of leiomyoma characteristics on quality of life at 36 months after UAE showed a correlation between fibroid size and poor symptom score outcome [9], while Spies et al. reported that patients with larger baseline fibroid volumes are three times more likely to show treatment failure one year post intervention [21]. On the other hand, several studies found no disadvantage for patients with large fibroids [16, 19, 29–31]. Bérczi et al. evaluated safety and effectiveness of UAE in fibroids larger than 10 cm and reported similar quality of life scores in both groups, respectively, thus concluding that there is no significant difference in the effectiveness or the complication rate between small and large fibroids [16]. Notably, a retrospective comparative study in 323 patients, who were instructed to express their symptomatic severity according to a ten-point visual analog scale, could not find any significant differences regarding symptom scores or in the presence of embolization-related complications between the control group and women with a large fibroid burden, defined as a dominant leiomyoma with a longest diameter of more than 10 cm or a uterine volume of at least 700 cm^3^ [30]. However, most of the abovementioned studies lack the use of standardized questionnaire and robust statistical analysis. Our study demonstrates a highly significant improvement in all UFS-QoL categories for both patient groups. When directly compared, our data demonstrates a significant better response in various UFS-QoL categories for women with small fibroids over short- and long-term observation periods using standardized and robust statistics. Interestingly, patients with small fibroids show lower median baseline scores, yet showing greater objective symptom improvement compared to women with large fibroids. A possible explanation for this could lay in the nature of large fibroids. Because of the slow growth of these tumors, some women might tend to better tolerate the symptoms or have learned to cope with them. These women would automatically look for a treatment later in the course of disease, hence presenting with larger fibroids. On the contrary, patients with small fibroids have only recently faced a reduction in HRQoL. Post-intervention, patients with small fibroids, thus, might seem to exhibit a positive overreaction as they quickly return to their previous level of HRQoL.

This study has several limitations. One limitation of this study is the chosen study design without randomization to a control group or another treatment method. All patients presented to us after having made the decision to undergo UAE and randomization was, consequently, not possible due to the lack of a control group. Moreover, UAE was performed by a group of board-certified radiologists, which might have resulted in differences in the therapeutic outcome because the end point of embolization is somewhat subjective and thus may vary between different interventionalists. Another limitation is the number of patients for subgroup analysis. Although the patient cohort is comparatively large, the number of patients was reduced due to lack of compliance regarding completion of the questionnaire. In addition, many patients were not reached in the long-term follow-up for questionnaire completion (‘lost to follow-up’). Finally, potential recall bias might exist as the timeframe of the long-term follow-up period was very heterogeneous with a span reaching from ten months to ten years.

## Conclusions

Based on the results presented in this study, we conclude that UAE is an efficient minimally invasive therapy for the treatment of symptomatic fibroids that is associated with a very low risk for re-interventions. Affected women benefit from a quick relief of fibroid-related symptoms as well as a marked improvement in HRQoL early after treatment. Patients with small fibroids tend to show a better response to UAE when compared to patients with large fibroids.

## Supplementary Information

**Additional file 1.** Full UFS-QoL questionnaire as well as the German translation.

## Data Availability

The datasets used and/or analyzed during the current study are available from the corresponding author on reasonable request.

## Abbreviations

CI: Confidence interval
FIBROID: Fibroid Registry for outcomes data
GDPR: General Data Protection Regulation
HRQoL: Health-Related Quality of Life
MRI: Magnetic resonance imaging
PVA: Polyvinyl alcohol
QR: Quartile range
TGM: Trisacryl gelatin microsphere
UAE: Uterine artery embolization
UFS-QoL: Uterine Fibroid Symptom and Quality of Life
