# Supplementary material for: Short- and long-term evaluation of disease-specific symptoms and quality of life following uterine artery embolization of fibroids
Source: Insights Imaging. 2022 Jun 21;13:106. doi: 10.1186/s13244-022-01244-1 (PMC9213618; doi:10.1186/s13244-022-01244-1)
Supplement: Supplementary file 1 — Additional file 1. Full UFS-QoL questionnaire as well as the German translation. [file 13244_2022_1244_MOESM1_ESM.docx]

**ELECTRONIC SUPPLEMENTARY MATERIAL**

**Short and long-term evaluation of disease-specific symptoms and quality of life following uterine artery embolization of fibroids**

**Supplement S1**


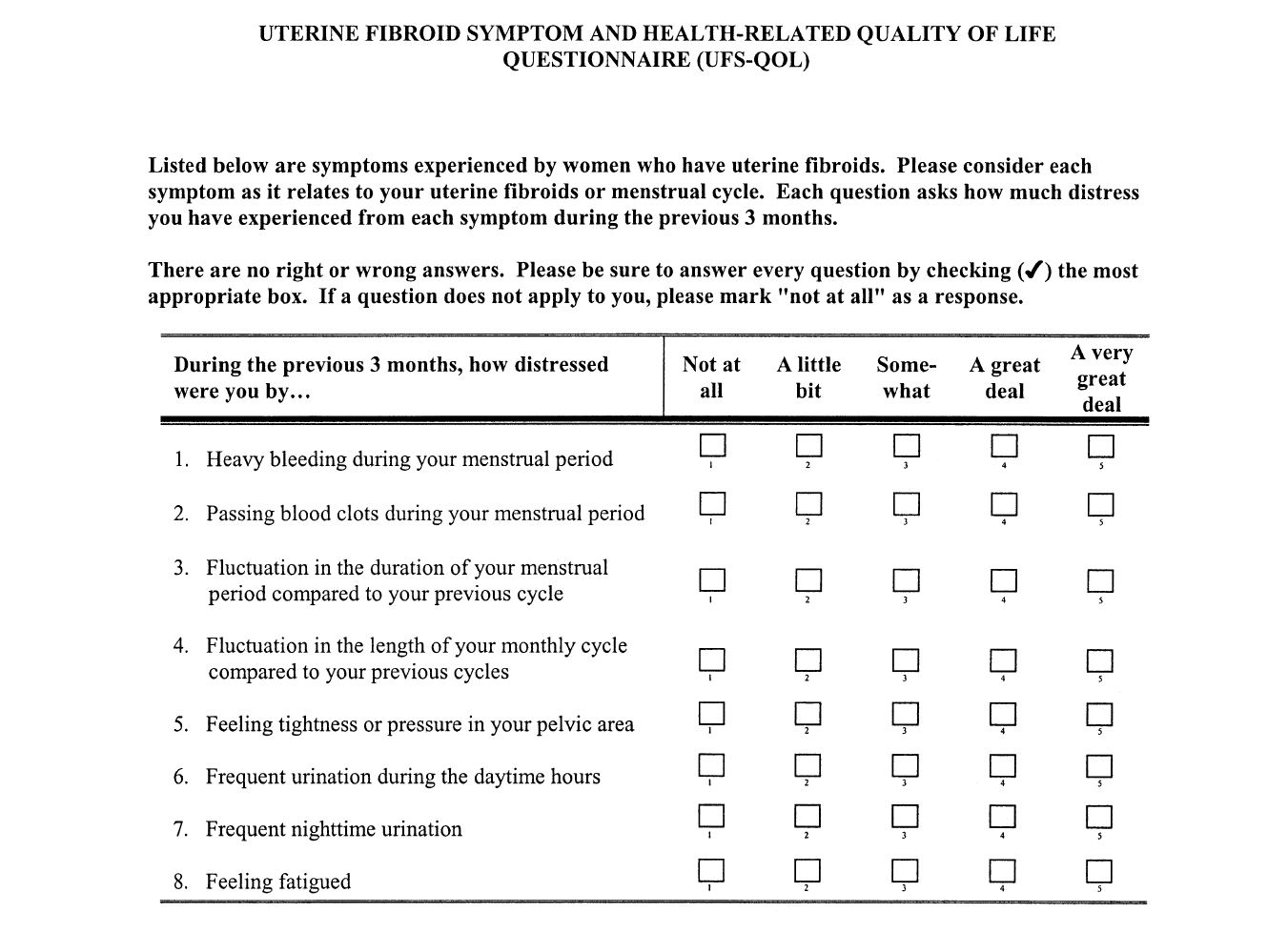


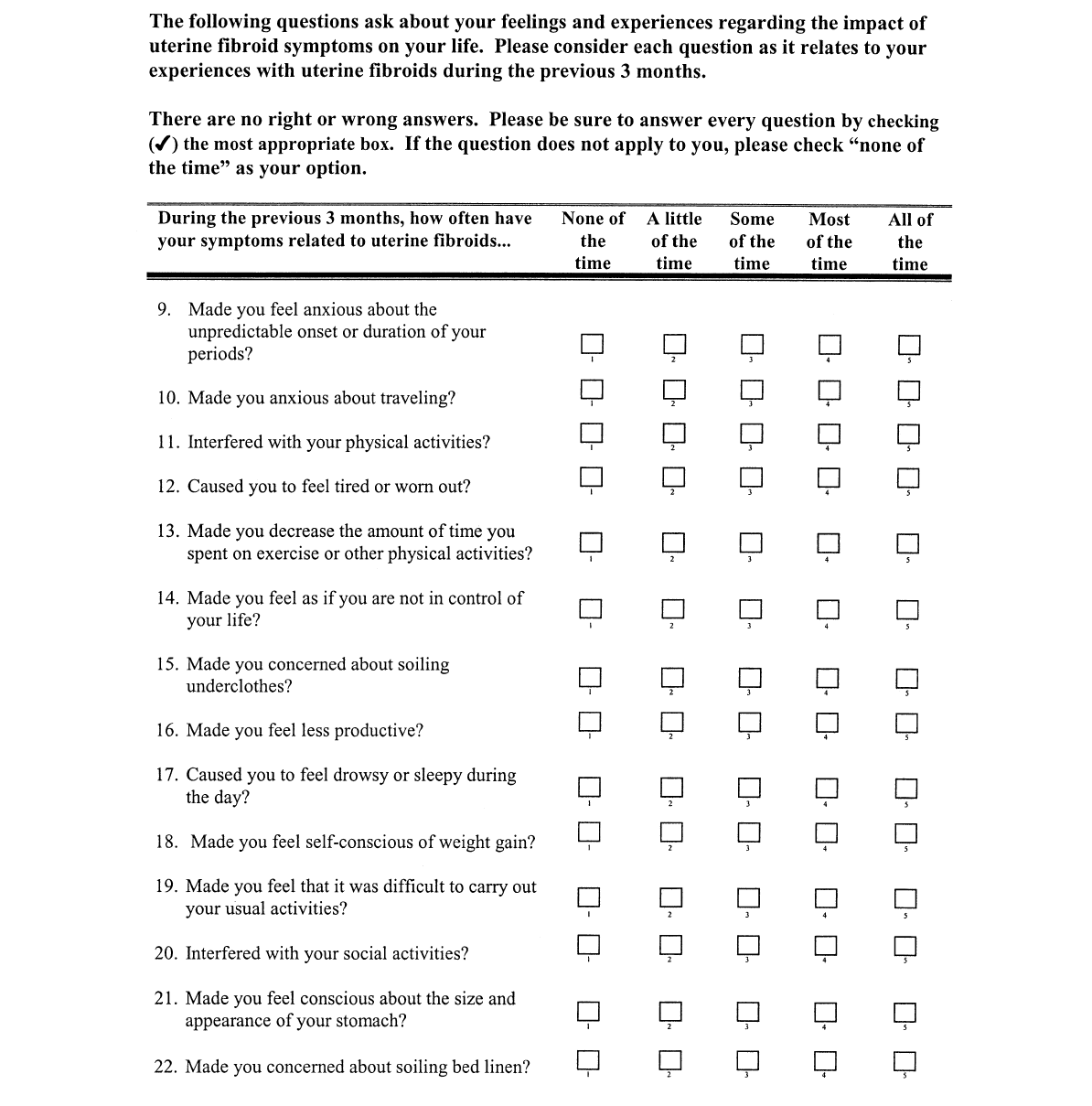


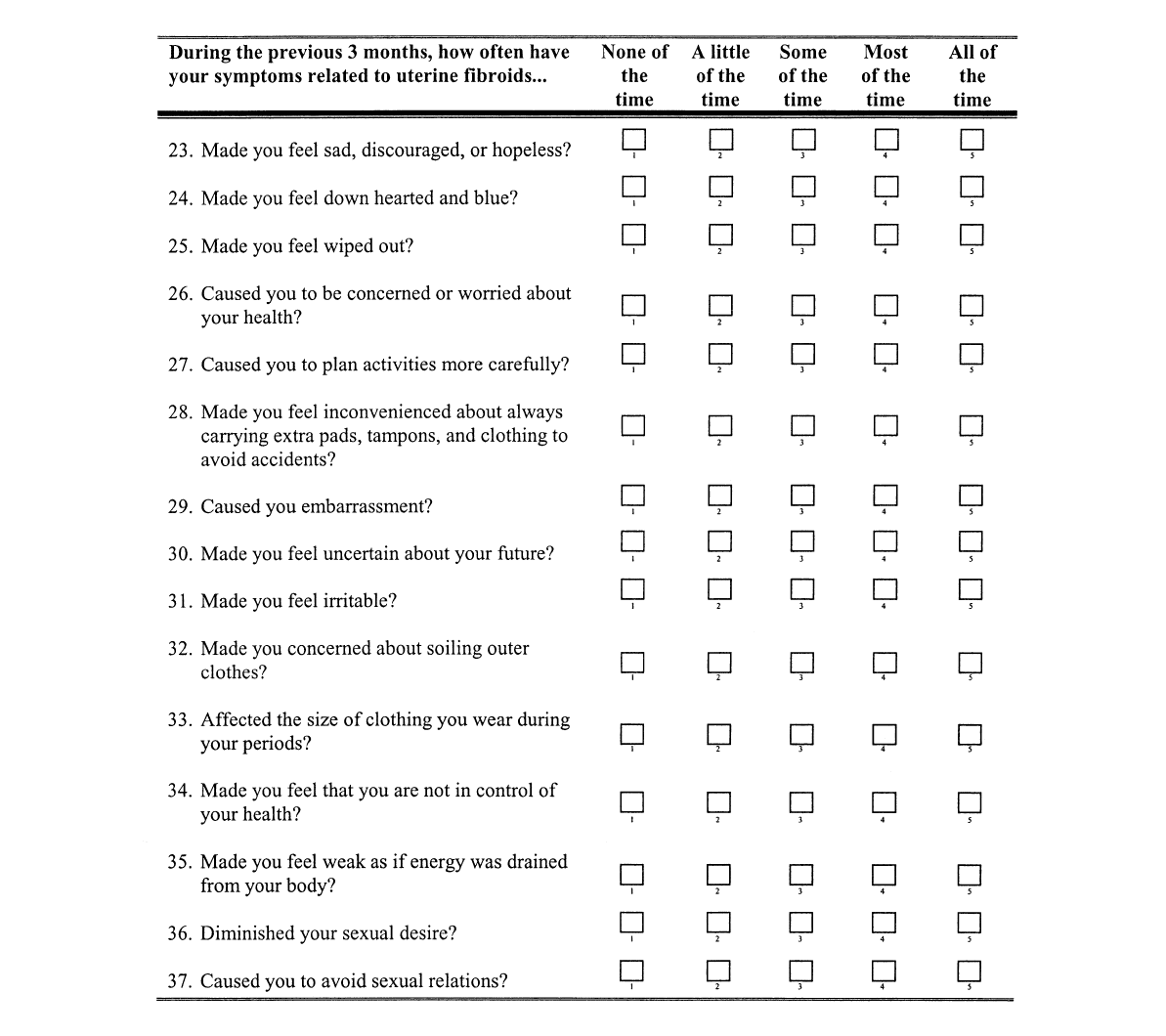


**Supplement S2**

**Nachstehend sind einige Beschwerden aufgeführt, die bei Frauen mit Gebärmuttermyomen auftreten können. Betrachten Sie bitte alle diese Beschwerden im Zusammenhang mit Ihren Gebärmuttermyomen oder Ihrer Monatsblutung. Diese Fragen sollen klären, wie sehr Ihnen diese Beschwerden in den letzten drei Monaten zu schaffen gemacht haben.**

Es gibt keine richtigen oder falschen Antworten. Bitte achten Sie darauf, dass Sie bei jeder Frage die Antwort ankreuzen (X), die am meisten auf Sie zutrifft. Wenn eine Frage nicht auf Sie zutrifft, kreuzen Sie bitte als Antwort "überhaupt nicht" an.

| **Wie sehr hat Ihnen in den letzten drei**  **Monaten folgendes zu schaffen gemacht:** | **Überhaupt nicht** | **Ein wenig** | **Mäßig** | **Ziemlich** | **Sehr** |
| --- | --- | --- | --- | --- | --- |
| 1. Starke Blutungen während Ihrer Periode | 1 | 2 | 3 | 4 | 5 |
| 2. Abgang von Blutgerinnseln während Ihrer Periode | 1 | 2 | 3 | 4 | 5 |
| 3. Schwankungen in der Dauer Ihrer Monatsblutung | 1 | 2 | 3 | 4 | 5 |
| 4. Unregelmäßigkeiten in Ihrem Monatszyklus | 1 | 2 | 3 | 4 | 5 |
| 5. Spannungs- oder Druckgefühl in der Beckengegend | 1 | 2 | 3 | 4 | 5 |
| 6. Häufiges Wasserlassen tagsüber | 1 | 2 | 3 | 4 | 5 |
| 7. Häufiges nächtliches Wasserlassen | 1 | 2 | 3 | 4 | 5 |
| 8. Müdigkeit | 1 | 2 | 3 | 4 | 5 |

**In den folgenden Fragen geht es um die Auswirkungen der Myombeschwerden auf Ihr Leben und um Ihre Gefühle und Ihre Erfahrungen damit. Bitte beziehen Sie jede Frage auf die Erfahrungen, die Sie in den letzten drei Monaten mit Ihren Gebärmuttermyomen gemacht haben.**

Es gibt keine richtigen oder falschen Antworten. Bitte achten Sie darauf, dass Sie bei jeder Frage die Antwort ankreuzen (X), die am meisten auf Sie zutrifft. Wenn eine Frage nicht auf Sie zutrifft, kreuzen Sie bitte als Antwort "nie" an.

| **Wie oft in den letzten drei Monaten haben die Beschwerden im Zusammenhang mit Ihren Gebärmuttermyomen...** | **Nie** | **Selten** | **Manchmal** | **Oft** | **Immer** |
| --- | --- | --- | --- | --- | --- |
| 9. dazu geführt, dass Sie sich Sorgen gemacht haben, weil der Beginn oder die Dauer Ihrer Monatsblutung nicht vorhersehbar waren? | 1 | 2 | 3 | 4 | 5 |

10. dazu geführt, dass Sie sich Sorgen

| gemacht haben, wenn Sie unterwegs sein wollten? | | 1 | | 2 | | 3 | 4 | | 5 |
| --- | --- | --- | --- | --- | --- | --- | --- | --- | --- |
| 11. Sie körperlich in dem, was Sie tun konnten, (z.B. Sport, spazieren gehen, …) beeinträchtigt? | | 1 | | 2 | | 3 | 4 | | 5 |
| 12. dazu geführt, dass Sie sich müde oder erschöpft gefühlt haben? | | 1 | | 2 | | 3 | 4 | | 5 |
| 13. dazu geführt, dass Sie weniger Zeit mit Sport oder anderen körperlichen Aktivitäten verbracht haben? | | 1 | | 2 | | 3 | 4 | | 5 |
| 14. dazu geführt, dass Sie das Gefühl hatten, Ihr Leben nicht im Griff zu haben? | | 1 | | 2 | | 3 | 4 | | 5 |
| 15. dazu geführt, dass Sie sich Sorgen darüber gemacht haben, dass Sie Ihre Unterwäsche beschmutzen könnten? | | 1 | | 2 | | 3 | 4 | | 5 |
| 16. dazu geführt, dass Sie das Gefühl hatten, weniger leistungsfähig zu sein? | | 1 | | 2 | | 3 | 4 | | 5 |
| 17. dazu geführt, dass Sie sich tagsüber schläfrig gefühlt haben? | | 1 | | 2 | | 3 | 4 | | 5 |
| 18. dazu geführt, dass es Ihnen unangenehm war, dass man Ihnen die Gewichtszunahme ansehen könnte? | | 1 | | 2 | | 3 | 4 | | 5 |
| 19. dazu geführt, dass es Ihnen schwer fiel, das zu tun, was Sie normalerweise tun? | 1 | | 2 | | 3 | | 4 | 5 | |
| 20. Ihre Kontakte und Unternehmungen mit anderen Menschen (z.B. ins Kino, in ein Restaurant oder auf eine Party gehen) beeinträchtigt? | 1 | | 2 | | 3 | | 4 | 5 | |
| 21. dazu geführt, dass Ihnen Größe und Aussehen Ihres Bauches peinlich waren? | 1 | | 2 | | 3 | | 4 | 5 | |

| **Wie oft in den letzten drei Monaten haben die Beschwerden im Zusammenhang mit Ihren Gebärmuttermyomen...** | **Nie** | **Selten** | **Manchmal** | **Oft** | **Immer** |
| --- | --- | --- | --- | --- | --- |
| 22. dazu geführt, dass Sie sich Sorgen darüber gemacht haben, dass Sie die Bettwäsche beschmutzen könnten? | 1 | 2 | 3 | 4 | 5 |
| 23. dazu geführt, dass Sie traurig, mutlos oder ohne Hoffnung waren? | 1 | 2 | 3 | 4 | 5 |
| 24. dazu geführt, dass Sie niedergeschlagen waren? | 1 | 2 | 3 | 4 | 5 |
| 25. dazu geführt, dass Sie sich völlig erschöpft fühlten? | 1 | 2 | 3 | 4 | 5 |
| 26. dazu geführt, dass Sie sich Sorgen über Ihre Gesundheit gemacht haben? | 1 | 2 | 3 | 4 | 5 |
| 27. dazu geführt, dass Sie sorgfältiger geplant haben, was Sie tun wollten? | 1 | 2 | 3 | 4 | 5 |
| 28. dazu geführt, dass es Ihnen lästig war, immer Ersatzbinden, Tampons oder Kleidung zum Wechseln bei sich zu haben, um „Missgeschicke“ zu vermeiden? | 1 | 2 | 3 | 4 | 5 |
| 29. dazu geführt, dass Sie in peinliche Situationen gerieten? | 1 | 2 | 3 | 4 | 5 |
| 30. dazu geführt, dass Sie sich Sorgen über Ihre Zukunft gemacht haben? | 1 | 2 | 3 | 4 | 5 |
| 31. dazu geführt, dass Sie gereizt waren? | 1 | 2 | 3 | 4 | 5 |

32. dazu geführt, dass Sie sich Sorgen

| darüber gemacht haben, dass Sie Ihre Oberbekleidung beschmutzen könnten? | 1 | 2 | 3 | 4 | 5 |
| --- | --- | --- | --- | --- | --- |
| 33. einen Einfluss darauf gehabt, wie weit die Kleidung war, die Sie während Ihrer Monatsblutung trugen? | 1 | 2 | 3 | 4 | 5 |
| 34. Ihnen das Gefühl gegeben, dass Sie  Ihre Gesundheit nicht im Griff haben? | 1 | 2 | 3 | 4 | 5 |
| 35. dazu geführt, dass Sie sich schwach fühlten, als würde Energie aus Ihrem Körper gezogen? | 1 | 2 | 3 | 4 | 5 |
| 36. Ihr sexuelles Verlangen gedämpft? | 1 | 2 | 3 | 4 | 5 |
| 37. dazu geführt, dass Sie sexuelle Betätigung vermieden haben? | 1 | 2 | 3 | 4 | 5 |
